# Identification of Potential Interacting Proteins With the Extracellular Loops of the Neuronal Glycoprotein M6a by TMT/MS

**DOI:** 10.3389/fnsyn.2020.00028

**Published:** 2020-07-23

**Authors:** Gabriela I. Aparicio, Karina Formoso, Antonella León, Alberto C. Frasch, Camila Scorticati

**Affiliations:** ^1^Instituto de Investigaciones Biotecnológicas, Universidad Nacional de San Martín (IIBio-UNSAM), Consejo Nacional de Investigaciones Científicas y Técnicas (CONICET), San Martín, Argentina; ^2^Instituto de Investigaciones Biomédicas (BIOMED), Facultad de Ciencias Médicas, Pontificia Universidad Católica Argentina (UCA), CONICET, San Martín, Argentina; ^3^Vicerrectorado, Edificio de Gobierno, Universidad Nacional de San Martín (UNSAM), San Martín, Argentina

**Keywords:** synaptic proteins, rat hippocampal neurons, mass spectometry, protein-protein interaction, proteolipd protein family

## Abstract

Nowadays, great efforts are made to gain insight into the molecular mechanisms that underlie structural neuronal plasticity. Moreover, the identification of signaling pathways involved in the development of psychiatric disorders aids the screening of possible therapeutic targets. Genetic variations or alterations in *GPM6A* expression are linked to neurological disorders such as schizophrenia, depression, and Alzheimer’s disease. *GPM6A* encodes the neuronal surface glycoprotein M6a that promotes filopodia/spine, dendrite, and synapse formation by unknown mechanisms. A substantial body of evidence suggests that the extracellular loops of M6a command its function. However, the proteins that associate with them and that modulate neuronal plasticity have not been determined yet. To address this question, we generated a chimera protein that only contains the extracellular loops of M6a and performed a co-immunoprecipitation with rat hippocampus samples followed by TMT/MS. Here, we report 72 proteins, which are good candidates to interact with M6a’s extracellular loops and modify its function. Gene ontology (GO) analysis showed that 63% of the potential M6a’s interactor proteins belong to the category “synapse,” at both sides of the synaptic cleft, “neuron projections” (51%) and “presynapse” (49%). In this sense, we showed that endogenous M6a interacts with piccolo, synaptic vesicle protein 2B, and synapsin 1 in mature cultured hippocampal neurons. Interestingly, about 28% of the proteins left were related to the “myelin sheath” annotation, suggesting that M6a could interact with proteins at the surface of oligodendrocytes. Indeed, we demonstrated the (*cis* and *trans*) interaction between M6a and proteolipid protein (PLP) in neuroblastoma N2a cells. Finally, the 72 proteins were subjected to disease-associated genes and variants screening by DisGeNET. Apart from the diseases that have already been associated with M6a, most of the proteins are also involved in “autistic disorder,” “epilepsy,” and “seizures” increasing the spectrum of disorders in which M6a could play a role. Data are available *via* *ProteomeXchange* with identifier *PXD017347*.

**GRAPHICAL ABSTRACT:**
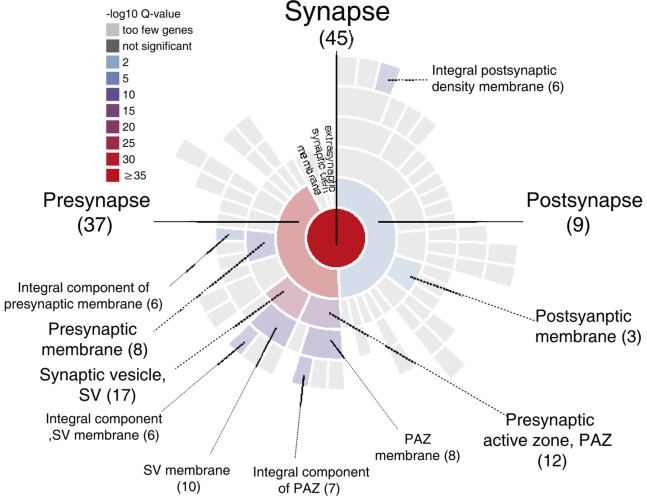
Sunburst plot from SynGO analysis of M6a’s potential synaptic interactors (https://www.syngoportal.org/, Koopmans et al., 2019). This work shows a combination of co-immunoprecipitation assay with quantitative tandem mass tag spectrometry (TMT/MS) to identify potential protein-protein interactions. Using a chimera protein that only contains the extracellular loops of neuronal glycoprotein M6a, we performed a co-IP with rat hippocampi samples followed by TMT/MS. Data analysis revealed 72 potential interactors of M6a’s loops, 45 of which are related to synapse localization.

## Introduction

The membrane glycoprotein M6a—together with proteolipid protein PLP1, DM20, and M6b—belongs to the tetraspan PLP family (Schweitzer et al., 2006). M6a is a neuronal surface protein that promotes neuronal stem cell differentiation, migration, neurite outgrowth, filopodia/spine induction, and synapse formation in primary neuronal cultures and (non-) neuronal cell lines (Alfonso et al., 2005; Michibata et al., 2008; Zhao et al., 2008; Michibata et al., 2009; Formoso et al., 2015a,b, 2016; Garcia et al., 2017; Honda et al., 2017). In humans, alterations in M6a levels or polymorphisms in *GPM6A*, are associated with depression, schizophrenia, claustrophobia, bipolar disorders, and learning disabilities (Boks et al., 2008; Greenwood et al., 2012; El-Kordi et al., 2013; Gregor et al., 2014; Fuchsova et al., 2015). In mice, variations in *Gpm6a* expression are linked to chronic stress/depression, claustrophobia, and Alzheimer’s disease (Alfonso et al., 2005; El-Kordi et al., 2013; Lachén-Montes et al., 2016). Nevertheless, the complete mechanism by which M6a participates in synaptic plasticity and how it is linked to disease onset remains unknown.

**Figure 1:**
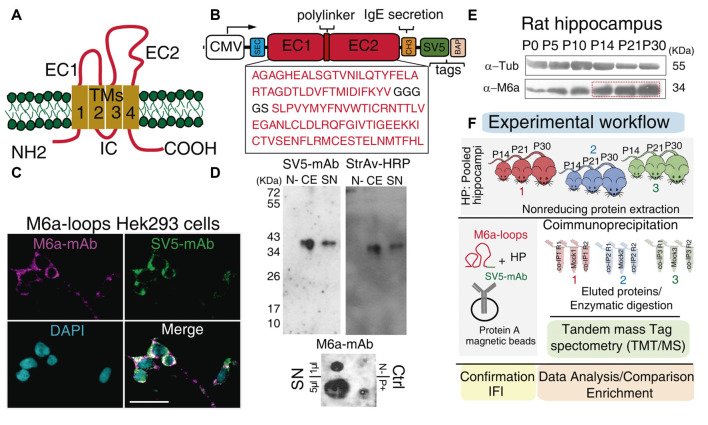
Workflow: from the chimera protein to co-immunoprecipitation and protein identification. **(A)** Schematic representation of M6a: it has four transmembrane domains (TM1-4), two extracellular loops, a small (EC1) and a large (EC2), an intracellular loop (IC) and the N- and C-terminus at the cell cytoplasm. **(B)** Scheme of the chimera protein: M6a-loops construct. The EC1 and EC2 of rat M6a (NCBI Reference Sequence: NP_835206.1), were cloned into the pBig plasmid. White box: CMV promoter, blue box: secretion signal peptide, SEC, red boxes: EC1, EC2, and the polylinker peptide, orange box: IgE secretion sequence, green box: SV5 tag and pink box: biotin acceptor peptide (BAP) tag. **(C)** M6a-loops-HEK293 cells were labeled with structural anti-M6a-mAb (magenta), anti-SV5-mAb (green), and the superposition of both channels is shown in white (Merge). Cells nuclei were stained with DAPI. Scale bar: 30 μm. **(D)** Representative Western blots (top panels) and Dot blot (DB, bottom panel) of cell extracts (CEs) and concentrated supernatants (SN) from M6a-loops-HEK293 cells and culture media respectively. Non-transfected HEK293 cells were used as the negative control. Rat hippocampi homogenates were used as a positive control in DB. Anti-SV5-mAb and Streptavidin-HRP were used to characterize the expression and secretion of M6a-loops. DB was done under non-denaturing conditions and 1 μl and 5 μl of M6a-loops from SN (0.5 mg/ml) were used. **(E)** Representative Western blot of rat hippocampus from postnatal day 0 (P0) to P30. Primary antibodies used were anti-M6a:COOH and anti-tubulin as the loading control. **(F)** Workflow of the co-IP-TMT/MS experiment. M6a-loops was captured with anti-SV5-mAb cross-linked into protein A magnetic beads. After, protein extracts from rat hippocampi homogenates of postnatal days 14, 21, and 30 (*n* = 3 animals per co-IP, one animal of each P day per experiment) were co-immunoprecipitated with M6a-loops. A total of three co-IPs were performed with their replicates (*n* = 9). Eluted proteins were enzymatically digested and peptides were subjected to TMT/MS. Finally, we performed data analysis, gene ontology (GO) enrichment analysis, and validation of these results.

According to topology predictions, M6a and PLP family members share structural similarity with the tetraspanin family, containing four transmembrane domains (TMs), two extracellular loops (EC1 and EC2), an intracellular loop (IC) and their N- and C-terminus facing the cell cytoplasm (Figure 1A). Tetraspanins are ubiquitous molecules involved in cell adhesion, migration, proliferation, and differentiation *via* cell-cell, matrix–cell and lateral associations in the plasma membrane. Each tetraspanin can interact with a group of partner proteins in a dynamic assembly forming tetraspanin-enriched microdomains (TEMs), which represent functional platforms (Yáñez-Mó et al., 2009; Scorticati et al., 2011). Thus, the specific tetraspanin functions in different cell types depending on their heterotypic association—within the TEMs—with different partner proteins. In this regard, a large number of different partner molecules might explain why tetraspanins are involved in such a wide variety of essential cellular processes. Mutagenesis studies of the extracellular loops of tetraspanins demonstrated that they are crucial for the specificity of protein-protein interactions. Particularly, the EC2 contains all of the known tetraspanin protein-protein interaction sites, and monoclonal antibodies that recognize cell surface epitopes so far exclusively recognize the EC2 domain. Whereas the EC1 is necessary for correct surface expression of the protein and it is responsible for EC2-interactor binding strength (Murru et al., 2018).

Regarding M6a, there is strong evidence suggesting that M6a’s extracellular loops contribute to its function. For instance, hippocampal neurons exposed to the monoclonal structural antibody (M6a-mAb), which recognizes M6a’s extracellular loops, arrests neurite extension and synapse formation (Lagenaur et al., 1992; Formoso et al., 2015a; Garcia et al., 2017). The M6a-mAb treatment induces M6a’s endocytosis and sorting to degradation compartments leading to plasticity impairment. Moreover, certain cysteine residues within the EC2 of M6a are functionally crucial sites for its folding and filopodia induction (Fuchsova et al., 2009). In summary, we and other groups proved the functional relevance of M6a’s extracellular loops in neuronal plasticity (Baumrind et al., 1992; Lagenaur et al., 1992; Mukobata et al., 2002; Fuchsova et al., 2009; Sato et al., 2011; Formoso et al., 2015a; Garcia et al., 2017). Hence, we hypothesize that the broad range of M6a’s functions depends on specific interactions through its extracellular loops in each particular cell and throughout each stage of development. Several proteins are involved in M6a mechanisms of action, such as protein kinase C (PKC), neuronal cell adhesion molecule (NCAM), Src family of tyrosine kinases, clathrin, ruffy-3, coronin-1A, N-methyl-D-aspartate receptor type1 (GRIN1), and synaptophysin. However, most of them are associated with M6a cytoplasmic tails (Mukobata et al., 2002; Scorticati et al., 2011; Fuchsova et al., 2015; Alvarez Juliá et al., 2016; Formoso et al., 2016; Garcia et al., 2017; Honda et al., 2017). Hence, the proteins that might interact with the extracellular loops of M6a and potentially control its function have not been determined yet. Here, to identify M6a’s extracellular loops partners, we designed and purified a chimera protein—containing both loops of M6a—and used it as bait in a co-immunoprecipitation assay (co-IP) followed by quantitative tandem mass tag spectrometry (TMT/MS; Figure 1F). Analysis of the 1,529 identified proteins revealed that 72 proteins, with a high degree of stringency, could be associated with M6a. Moreover, functional enrichment analysis by gene ontology (GO) showed that most proteins are involved in neuronal plasticity at the synapse level. We were able to confirm three proteins (piccolo, synapsin 1, and synaptic vesicle glycoprotein 2B) which colocalized with M6a at the synaptic boutons and may have a crucial role in M6a-induced plasticity. Interestingly, we report that M6a can interact with PLP, suggesting a new role of M6a in neuron-glia interaction. Moreover, we interrogated the DisGeNET database and most of the enriched proteins were associated with depression, schizophrenia, bipolar disorder, and Alzheimer’s disease.

## Materials and Methods

### Animals

Pregnant Sprague–Dawley rats maintained at the Facultad de Farmacia y Bioquímica of the Universidad of Buenos Aires (FFyB-UBA) were used. All animal procedures were carried out according to the guidelines of the National Institutes of Health (publications No. 80-23) and approved by the Committee for the Care and Use of Laboratory Animals of the Universidad Nacional de San Martín (CICUAE-UNSAM).

Primary cultures of hippocampal neurons were prepared from fetal rats at embryonic day 19 (CICUAE-UNSAM No. 03/2015, see also “Cell Culture”). On the other hand, litters from two female Sprague–Dawley rats were used for co-IP experiments. The pups were housed with their mother in individual cages until weaning on day 21. The animals were cared for according to the protocol of CICUAE-UNSAM No. 03/2016. During all experiments, animals were kept at the IIBio-UNSAM lab animal facility. All animals had free access to food and water and were maintained in a 12/12 h dark/light cycle. The pup’s age was determined as life-days after birth, the day of birth was considered as postnatal day zero (P0) and the subsequent days as P1 to P30. The litters were randomly allocated in three independent co-IP experiments.

### Reagents and Antibodies

Primary antibodies were: monoclonal anti-M6a rat IgG (M6a-mAb, 1/1,000; Medical and Biological Laboratories, MBL, Nagoya, Japan), polyclonal rabbit anti-C terminus of M6a (anti-M6a:COOH, 1/750) developed in our laboratory (Scorticati et al., 2011), monoclonal mouse anti-alpha-tubulin (anti-Tub, 1/1,000; Sigma, Munich, Germany), monoclonal mouse anti-SV5 tag IgG_2a_ (anti-SV5-mAb, 1/1,000; Invitrogen, Leiden, Netherlands), polyclonal rabbit anti synapsin 1 (anti-Syn1, 1/500; Chemicon International, Temecula, CA, USA), monoclonal mouse anti-human transferrin receptor (anti-TfR, 1/500; Zymed Laboratories, San Francisco, CA, USA), polyclonal rabbit anti synaptic vesicle protein 2B (anti-SV2B, 1/200), polyclonal rabbit anti piccolo (anti-PCLO, 1/200; Synaptic Systems, Göttingen, Germany). Secondary antibodies were: rhodamine-conjugated goat anti-rat IgG (H + L; 1/1,000; Jackson ImmunoResearch Laboratories, West Grove, PA, USA) and preabsorbed secondary antibodies: Alexa 488 goat anti-rabbit IgG (H + L; 1/1,000), Alexa 488 goat anti-mouse IgG (H + L; 1/1,000; Invitrogen, Oregon, USA). Antibodies HRP-conjugated were: polyclonal goat anti-rabbit IgG (1/16,000; Sigma, St. Louis, MO, USA) and polyclonal goat anti-mouse IgG (1/5,000; Sigma, Saint Louis, MO, USA).

Other reagents used were: 4′,6-Diamidino-2-phenylindole dihydrochloride (DAPI, Sigma), biotin (Sigma), streptavidin-HRP conjugated (BD Bioscience, San Jose, CA, USA), protein A/G-HRP conjugated (Thermo Fisher Scientific, Waltham, MA, USA), protease inhibitor cocktail (PIC, Sigma), lipofectamine LTX with Plus reagent (Thermo Fisher Scientific, Waltham, MA, USA), trypsin, sequencing grade (Promega, Fitchburg, WI, USA) and paraformaldehyde 16% solution (PFA; Electron Microscopy Sciences, Hatfield, PA, USA).

### Plasmids

To identify proteins that could interact with the extracellular loops of M6a, we designed a chimera protein called M6a-loops. The amino acid sequence for M6a-loops contains the small (EC1, 44-84) and large (EC2, 149-213) extracellular loops of rat M6a (NCBI Reference Sequence: NP_835206.1), both linked by a linker peptide (GGGGS). A secretion sequence, SEC, was fused to the N-terminus of the EC1 according to (Predonzani et al., 2008). At the C terminus of the EC2, the human IgG heavy chain constant domain 3 (CH3) sequence was added, as this sequence was described as an enhancer of secretion of recombinant proteins (Poggianella et al., 2015). The final DNA sequence of M6a-loops was synthesized by GeneScript and cloned into the HindIII and KpnI sites of the pUC18 plasmid. Then, the M6a-loops sequence was sub-cloned into the pBig-BirA-SV5-biotin acceptor peptide (BAP; pBig) plasmid (Predonzani et al., 2008). pBig, a single bigenic plasmid, has two different and independent gene cassettes. The first one codifies the sec-BirA enzyme, which recognizes and biotinylates a BAP. The second cassette has two tags: an Sv5 tag sequence and the BAP sequence. M6a-loops sequence was inserted into the second cassette of the pBig plasmid, upstream the Sv5 tag sequence (Figure 1B).

For green fluorescent protein (GFP) or red fluorescent protein (RFP)-tagged proteins, a plasmid encoding for GFP/RFP (EGFP-C1 and RFP-C1; Clontech Laboratories, Palo Alto, CA, USA) fused in frame with the coding sequence of mouse PLP and mouse M6a were used (Fernández et al., 2010; Formoso et al., 2015a). GFP-tagged transferrin (Trf-GFP) was kindly supplied by Dr. Juan S. Bonifacino’s lab (NIH, Bethesda, MD, USA; Rodriguez-Walker et al., 2015).

### Cell Culture and Plasmid Transfection

#### Hippocampal Cultures

Dissociated neuronal cultures were prepared from rat hippocampi of embryonic day 19, as previously described (Formoso et al., 2015a). Briefly, tissues were treated with 0.25% trypsin in Hanks’ solution at 37°C for 15 min. A single-cell solution was prepared in Neurobasal^®^ medium (NB, Invitrogen) containing 2 mM glutamine (Sigma), 1 mg/ml gentamicin (Sigma) with 10% (v/v) horse serum (Gibco^®^, Thermo Fisher Scientific, Waltham, MA, USA). Cells were seeded on coverslips coated with 0.1 mg/ml poly-L-lysine hydrobromide (Sigma) and 20 mg/ml laminin (Invitrogen) at a low density of 7,000 cells per well into a 24-well plate. After 2 h, the medium was changed to NB (NB1X with 1 g/L ovalbumin and B27 serum-free supplements from Invitrogen). Neurons were cultured for 12–15 days (DIV) and based on morphological characteristics, we estimated that more than 90% of the cells in the cultures were neurons.

#### Cell Lines

Human embryonic kidney cells (HEK293) cells were cultured in Dulbecco’s modified Eagle’s medium (DMEM, Gibco) supplemented with high glucose 0.35% (m/v), 200 mM L-alanine L-glutamine (GlutaMAX, Invitrogen), 10% (v/v) fetal bovine serum (FBS, Gibco) and 1 mg/ml gentamicin. Stable M6a-loops-HEK293 clones were selected with 0.4 mg/ml G418 (Sigma) according to protocols described in Wurm (2004).

For colocalization assays, murine neuroblastoma N2a cells were cultured in DMEM with 20% (v/v) FBS and 1 mg/ml gentamicin. Cells were seeded on coverslips in 24-well plates and then transiently co-transfected with M6a-GFP/RFP and with PLP-GFP or Tfr-GFP with Lipofectamine LTX with Plus reagent according to the manufacturer’s instructions (Thermo Fisher Scientific, Waltham, MA, USA).

### Protein Expression and Purification

Stable M6a-loops-HEK293 cells were cultured and maintained as described, in a T175 flask until confluence. Then, the culture medium was replaced with a serum-free medium supplemented with 100 mM biotin (Sigma). After 72 h, culture supernatant (SN) and CEs were collected. SN samples, having M6a-loops in solution, were filtered with a 0.45 μm filter and then concentrated to a final volume of 1.0–1.5 ml by consecutive rounds of centrifugation at 1,500 *g* for 40 min at 4°C using a Centripep, centrifugal filter device (3 kDa pore membrane, Amicon-Millipore Corporation). Then, SN’s protein concentration was measured by NanoDrop One (Thermo). CE samples were collected with a scrapper, centrifuged at 1,500 rpm for 5 min, and re-suspended in 150 μl of Triton X-100 0.1% in PBS with PIC. Then, CE samples, having a retained fraction of M6a-loops, were sonicated and pelleted by centrifugation (15,000 *g* for 10 min at 4°C).

### Immunocytochemistry

For endogenous M6a staining, cells (M6a-loops-HEK293 cells and hippocampal neurons) were incubated for 1 h at 4°C with anti-M6a mAb (1 mg/ml) in fresh medium. Afterward, cells were washed with PBS and labeled with rhodamine-conjugated goat anti-rat IgG (H + L; 1/1,000) for 1 h at 4°C. Then, cells were fixed in 4% paraformaldehyde and 4% sucrose in PBS at room temperature for 10 min. Next, cells were permeabilized with 0.1% Triton X-100 in PBS for 5 min and blocked with 3% bovine serum albumin (BSA) in PBS for 1 h. Cells were labeled with primary antibodies in 3% BSA for 16 h at 4°C. The next day, cells were labeled with preabsorbed secondary antibodies: Alexa fluor 488 goat anti-mouse or Alexa fluor 488 goat anti-rabbit (1/1,000) for 1 h at room temperature. Then, nuclei were stained with DAPI (1/5,000) for 5 min at room temperature. Coverslips were mounted in Fluorsave ^®^ (Calbiochem).

In the case of M6a-RFP and PLP-GFP or TfR-GFP expressing cells, the coverslips were washed, fixed, and mounted.

### Image Analysis

For characterization of the chimera protein, M6a-loops-HEK293 cells were imaged with a 1.4 NA, 60× objective lens on a Nikon Eclipse TE2000 inverted microscope coupled to an ORCA II ER CCD camera controlled by Metamorph 6.1 software. For colocalization analysis, cells were imaged at a 60× objective lens with a numeric aperture of 1.42 on an Olympus FV1000 confocal microscope. We set up the Olympus Fluoview v3.1a software to acquire the images with a 4–10 μs/pix of dwell time. We manually adjusted the laser energy setting (HV, gain and offset) by using slides stained only with the secondary antibodies to determine the threshold of background signal, which was applied to each image of the experiment. For colocalization cell images were in raster scan mode satisfying the Nyquist criterion, pixel size was 2–3 times smaller than the object.

The colocalization of puncta between M6a and pre-synaptic markers (piccolo, SV2B, and synapsin 1) was assessed in approximately 10–15 neurons per condition. The selected neurons were at least two cell diameters away from their nearest neighbor. The colocalization of puncta was determined using the plugin Puncta Analyzer from ImageJ (NIH) as previously described (Ippolito and Eroglu, 2010; Formoso et al., 2016). Briefly, three regions of interest (25 μm of dendrite length) from each neuron were selected, and then the background was subtracted. The threshold was adjusted manually for each channel. The minimum puncta size was set to four pixels. For each neuron analyzed, the number of puncta colocalization of the three segments were measured and averaged. Data are expressed as the average of synaptic puncta ± SEM. Three independent experiments were performed.

Estimation of colocalization between M6a and PLP or TfR was performed with the Coloc2 plugin of ImageJ. Coloc2 calculated Pearson’s correlation coefficient above the threshold for each pair of proteins and ranges between −1 and 1. Pearson’s coefficient higher than 0.5 was considered positive for colocalization. Mander’s coefficients above the threshold M1 (for channel 1, green) and M2 (for channel 2, red) were quantified for those pairs with a Pearson’s greater than 0.5 in the selected region of interest (three ROIs per cell). The average of each Mander’s coefficient was calculated and plotted. M1 and M2 estimate the fluorescence intensity of the two colors that overlap in a pixel from the PLP or TfR image to M6a and vice versa, respectively. A coefficient close to one indicates a high overlap of the signals (Formoso et al., 2015b). Data are expressed as the average of Pearson’s coefficient ± SEM.

Images were processed in Adobe Photoshop (version 8.0.1; Adobe Systems, San Jose, CA, USA).

### Western Blotting

Supernatant concentrated samples and whole-cell lysates were processed in the presence of PIC. Samples containing an equal amount of protein from rat hippocampi at different postnatal age, SN, and CE were analyzed under reducing conditions in a 10% SDS-PAGE. After electrophoresis, proteins were transferred onto a nitrocellulose membrane (Millipore) in a tank blot apparatus (Bio-Rad Laboratories). Membranes were blocked in Tris-buffered saline (TBS) solution containing 5% non-fat dried milk for 1 h at room temperature and incubated with primary antibodies diluted in 1% BSA-PBS overnight at 4°C. The next day, membranes were washed with TBS-T (TBS–0.2% Tween 20) and incubated with HRP-conjugated antibodies for 2 h at room temperature. To detect biotinylated M6a-loops, membranes were blocked overnight with 5% BSA-TBS at 4°C. The next day, membranes were incubated with streptavidin-HRP for 2 h at room temperature. Antigen-antibody complexes were detected according to a standard Enhanced Chemiluminescence blotting protocol using Super Signal Chemiluminescent Substrate (ECL-Pierce) and CL—Xposure films (Thermo Fisher Scientific, Waltham, MA, USA).

### Dot Blotting

To determine whether M6a-loops in SN samples were correctly folded, we performed a dot blot (DB) assay under native conditions. Two spots of 1 μl and 5 μl of SN (0.5 mg/ml) were placed on a PVDF membrane (Hybond-P, Amersham Life science), activated according to the manufacturer’s instructions. Non-specific sites were blocked with 1% BSA diluted in TBS for 1 h at room temperature. Afterward, membranes were incubated with anti-M6a-mAb overnight at 4°C. The next day, the membrane was washed three times with 0.1% BSA diluted in TBS for 5 min each and incubated with anti-G protein HRP conjugated for 1 h at room temperature. The antigen-antibody complexes were detected as described above.

### Co-immunoprecipitation

Three independent co-IPs, between M6a-loops and pooled rat hippocampi (HP), were performed. Briefly, nine hippocampi from P14 (*n* = 3), P21 (*n* = 3), and P30 (*n* = 3) rats were dissected and resuspended separately in 100 μl of Triton X-100 1% in PBS with PIC. Then, the nine samples were sonicated and pelleted by centrifugation (15,000 *g* for 10 min at 4°C). For each independent co-IP, each pool was composed of a combination of hippocampi from the three different ages. In other words, a total of nine individuals were sacrificed at different postnatal days, split and pooled in three different Co-IP samples. Pooled hippocampi samples were clarified (HPc) using 200 μl of protein A magnetic beads (SureBeads^TM^, BioRad) for 2 h at room temperature under rotation. Then, two conditions were tested: a mock condition (Mock_1–3) and the co-IP condition (co-IP 1–3, with two experimental replicates R1–2). For the co-IPs, the anti-SV5 tag-mAb was chemically cross-linked to protein A magnetic beads. Then, 10–25 μg of M6a-loops obtained from SN samples were incubated with the anti-SV5-magnetics beads (co-IP) or with protein A magnetic beads only (Mock) for 1 h at room temperature under rotation. Afterward, each condition was incubated with HPc (200–350 μg) overnight at 4°C under rotation. The table below summarizes each condition and the reagents used for each co-IP assay.

On the next day, samples were washed three times with PBS-T, and protein complexes were eluted with 50 μl of SDS-buffer with dithiothreitol (DTT) 10 mM and boiled for 10 min.

### Sample Preparation and Mass Spectrometry Data Acquisition and Analysis

#### Sample Preparation and TMT Labeling

The nine final samples were sent to the Proteomic Core Facility of the European Molecular Biology Laboratory (EMBL, Heidelberg). The reduction of disulfide bridges in cysteine-containing proteins was performed with 10 mM DTT diluted in 50 mM HEPES, pH 8.5 for 30 min at 56°C. Reduced cysteines were alkylated with 20 mM 2-chloroacetamide diluted in HEPES for 30 min at room temperature in the dark. Samples were prepared using the SP3 (Hughes et al., 2019). Digestion of samples was done with trypsin, in an enzyme to protein ratio 1:50 overnight at 37°C. The next day, peptide recovery was done in HEPES buffer by collecting supernatant on the magnet and combining with the second elution wash of beads with HEPES buffer.

**Table:** 

Co-immunoprecipitation assay						
	Samples	Protein A magnetic beads	anti-SV5 tag batch	M6a-loops	HPc
1	Mock 1	50 μl	X	20–25 μg/each	Pool 1: 350 μg/each
	co-IP1 R1	50 μl	7, 5 μg/Batch #1905424
	co-IP1 R2	50 μl
2	Mock 2	50 μl	X	10–15 μg/each	Pool 2: 200 μg/each
	co-IP2 R1	50 μl	5 μg/Batch #1222254
	co-IP2 R2	50 μl
3	Mock 3	50 μl	X	10–15 μg/each	Pool 3: 350 μg/each
	co-IP3 R1	50 μl	5 μg/Batch #1905424
	co-IP3 R2	50 μl

Peptides were labeled with TMT10plex Isobaric Label Reagent (ThermoFisher) according to the manufacturer’s instructions (Werner et al., 2014). Briefly, 0.8 mg of TMT reagent was dissolved in 42 μl acetonitrile (100%) and 4 μl of stock was added and incubated for 1 h at room temperature. Then, the reaction was quenched with 5% hydroxylamine for 15 min and the digested co-IP samples were combined for the TMT10plex. For further sample clean up, an OASIS^®^ HLB μElution Plate (Waters) was used. Offline high pH reverse phase fractionation was carried out on an Agilent 1200 Infinity high-performance liquid chromatography system, equipped with a Gemini C18 column (3 μm, 110 Å, 100 × 1.0 mm, Phenomenex).

#### Mass Spectrometry Data Acquisition

An UltiMate 3000 RSLC nano-LC system (Dionex) fitted with a trapping cartridge (μ-Precolumn C18 PepMap 100, 5 μm, 300 μm i.d. × 5 mm, 100 Å) and an analytical column (nanoEase^TM^ M/Z HSS T3 column 75 μm × 250 mm C18, 1.8 μm, 100 Å, Waters) was used. Samples were trapped with a constant flow of 0.1% formic acid in water (Solvent A) at 30 μl/min onto the trapping column for 6 min. Subsequently, peptides were eluted *via* the analytical column with a constant flow of 0.3 μl/min with an increasing percentage of 0.1% formic acid in acetonitrile (Solvent B) from 2% to 4% in 4 min, from 4% to 8% in 2 min, then 8% to 28% for a further 96 min, and finally from 28% to 40% in another 10 min. The outlet of the analytical column was coupled directly to a QExactive plus (Thermo Fisher Scientific, Waltham, MA, USA) mass spectrometer using the Proxeon nano-flow source in positive ion mode.

After, peptides were introduced into the QExactive plus *via* a Pico-Tip Emitter 360 μm OD × 20 μm ID; 10 μm tip (New Objective), and an applied spray voltage of 2.3 kV. The capillary temperature was 320°C. The full mass scan was acquired with a mass range of 375–1,200 m/z in profile mode with a resolution of 70,000. The filling time was set at a maximum of 10 ms with a limitation of 3 × 10^6^ ions. Data-dependent acquisition (DDA) was performed with the resolution of the Orbitrap set to 35,000, with a fill time of 120 ms and a limitation of 2 × 10^5^ ions. The normalized collision energy of 32 was applied and the dynamic exclusion time of 30 s was used. The peptide match algorithm was set to “preferred” and charge exclusion “unassigned,” charge states 1, 5–8 were excluded. MS^2^ data were acquired in profile mode.

#### MS Data Processing

The raw data were acquired and processed with IsobarQuant and Mascot (v2.2.07) respectively (Franken et al., 2015). The data was processed against the Uniprot *Rattus norvegicus* proteome database (ID: UP000002494, downloading date: 05142016, number of entries: 31673, including common contaminants, keratins and reversed sequences). The following modifications were included in the search parameters: Carbamidomethyl (C) and TMT10 (K; fixed modification), Acetyl (Protein N-term), Oxidation (M) and TMT10 (N-term; variable modifications). For the full scan (MS1), a mass error tolerance of 10 ppm and for MS/MS (MS2) spectra of 0.02 Da was set. For protein identification, these parameters were also required: trypsin as protease (with an allowance of maximum two missed cleavages); seven amino acids as minimum peptide length and at least two unique peptides for the same protein. The false discovery rate (FDR) on peptide and protein level was set to 0.05.

#### Differential Protein Analysis

The protein.txt output files of IsobarQuant were processed with the R programming language (R Development Core Team, 2008). To ensure data quality, only proteins that were quantified with at least two unique peptides were considered for the analysis. In total, 1,529 proteins passed this quality step. The TMT reporter ion intensities (signal.sum columns) were first cleaned for batch-effects using the “removeBatchEffect” function of the Limma package and further normalized using variance stabilization normalization (Huber et al., 2002). Different normalization coefficients were estimated for the “mock” condition to account for the lower protein amount. Proteins were tested for differential expression with a moderated *t*-test using the Limma package. The replicate factor was included in the linear model. A protein was annotated as a “hit” with a fold-change of at least 2-fold and an FDR below 0.05 and annotated as a “candidate” with a fold-change of at least 1.5-fold and an FDR below 0.2.

### Gene Ontology Analysis

The list of identified proteins was manually-curated based on literature (Végh et al., 2014; Jacquemet et al., 2019; Lleó et al., 2019) and their subcellular localization according to the UniProt database^1^. Proteins from the nucleus, cytosol, organelles, and membrane proteins from the cytosolic side of the cell were excluded. Proteins secreted, established in the extracellular matrix, plasma membrane and synaptic vesicles were selected for the enrichment analysis. GO enrichment and gene prioritization were made using the ToppFun application of ToppGene Suite server^2^ (Chen et al., 2009).

### Statistical Analysis

All data were processed with the R programming language (R Development Core Team, 2008)^3^. For differential protein expression analysis between conditions, a *t*-test was used and applied with Linear Models for Microarray Data (Limma) package (Ritchie et al., 2015). Two-tailed Pearson correlation coefficient (r) between biological replicates of total identified proteins per co-IP was calculated. Considering positive and significant correlation when *p* < 0.05.

## Results

### Design and Characterization of a Recombinant Bait Protein

Co-immunoprecipitation in combination with mass spectrometry is a powerful tool to identify potential protein-protein interactions (Sommer et al., 2014). Thus, to elucidate endogenous proteins that could potentially interact with M6a’s extracellular loops we first cloned a chimera protein containing both extracellular loops of M6a (M6a-loops) in a bigenic plasmid (pBig). The pBig plasmid has two different and independent gene cassettes: one for the sec-BirA enzyme (which biotinylates proteins in a specific amino acid sequence) and the other for the target protein. Figure 1B shows a schematic representation of the fusion protein cloned into pBig plasmid: M6a-loops is followed by the secretory peptide sequence of IgE (CH3), SV5 tag, and the BAP (Predonzani et al., 2008). We first determined whether M6a’s loops could be expressed and folded properly in the chimera protein. For that purpose, M6a-loops expressing HEK293 cells were incubated with a structural M6a-mAb antibody for 45 min at 4°C. Afterward, the cell surface was labeled with secondary rhodamine-conjugated antibodies (shown in magenta) and then fixed, permeabilized, blocked, and labeled with an anti-SV5-mAb antibody (shown in green). Figure 1C shows the binding features of M6a-loops in the cell surface of stable M6a-loops-expressing cells (M6a-loops-HEK293 cells). An overlap of both magenta and green signals was observed all along the cell surface, demonstrating that our recombinant protein was properly folded and expressed the SV5 tag. The staining pattern of non-transfected HEK293 cells is shown in Supplementary Figure S1. Concentrated supernatants-SN- and cell extracts-CE- from M6a-loops-HEK293 cells were then analyzed by immunoblots. Streptavidin-HRP tagged and anti-SV5-mAb antibodies were used to verify whether both samples express the chimera. Figure 1D shows a representative Western blot in which a unique band of approximately 36 kDa was recognized by anti-SV5-mAb and streptavidin-HRP in both CE and SN. Intact HEK293 cells were used as a negative control.

As we mentioned, M6a-mAb is a structural monoclonal antibody that recognizes conformational epitopes at M6a’s extracellular loops (Lagenaur et al., 1992). Thus, we performed a DB under non-denaturing conditions with SN samples from M6a-loops-HEK293 cells (Figure 1D, bottom panel). We also used SN samples from intact HEK293 cells and homogenates of rat hippocampus as negative and positive controls respectively. A positive signal was observed with 1 μl and 5 μl of SN (0.5 mg/ml) from M6a-loops-HEK293 cells, which indicates that M6a-loops was correctly folded in the SN samples. Non-denatured hippocampus sample showed a positive dot spot compared with the negative sample from intact HEK293 cells. In summary, we successfully generated a stable cell line that expresses and secretes a biotinylable chimera protein, M6a-loops, tagged with SV5, and recognized by the structural M6a-mAb.

The following step was to obtain the best sample in which to perform the co-immunoprecipitation. The level of proteins involved in synapse and dendritic spine formation varies during extra-uterine brain development (Penzes et al., 2013). It is well known that glycoprotein M6a exhibits strong expression in the rat hippocampal formation in association with synapse development and maintenance (Cooper et al., 2008; Formoso et al., 2016; Garcia et al., 2017). Thus, we first assessed whether rat hippocampus M6a expression changes from birth (postnatal 0, P0) up to adulthood (P30). Samples of each condition were subjected to SDS-PAGE followed by Western blotting using an anti-M6a antibody, which recognizes the C-terminus of the protein, and anti-tubulin as a loading control. As it can be observed in Figure 1E, M6a expression increases during hippocampus development and exhibits a maximum between P14-P30.

### Proteomics Analysis

For the proteomic study, three independent co-IP experiments were performed as described in the materials and methods section. For hippocampi sampling, we took into consideration two main aspects: the intrinsic variability between different animals and ages and the minimum amount of protein needed to perform co-IP experiments (van der Geer, 2014; Bonifacino et al., 2016). Hence, for each co-IP assay, nine pups from P14, P21, and P30 were sacrificed and hippocampi were collected and pooled (HP, see “Material and Methods” section). Then, HP samples were clarified and subjected to three independent co-IPs using M6a-loops captured with anti-SV5-mAb in the surface of protein A magnetic beads. HPc samples incubated with M6a-loops and protein A magnetic beads were used as Mock conditions. All samples were eluted and then subjected to tandem mass spectrometry identification (TMT/MS). Protein digestion, TMT labeling, fractioning and protein identification were performed for whole samples in the same run to minimize inter-assay variation (Figure 1F).

**Figure 2:**
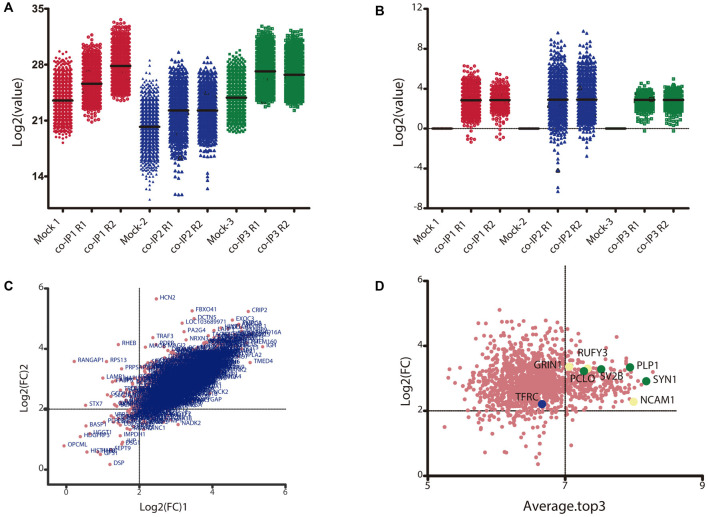
Data transformation and analysis of differentially immunoprecipitated proteins per condition.** (A)** Raw intensity signal data from the TMT/MS assay. The Log_2_ of the raw intensity signal data is represented for each identified protein in each control (Mock 1-3) and experimental condition with their replicate (co-IP1-3R1-2). **(B)** Raw signal data after batch effect removal and data transformation. The plot shows the Log_2_ of the control ratio for each protein identified in each experimental condition after being processed. **(C)** Correlation between replicates for each co-IP experiment. The plot shows the Log_2_ of the fold change (Log_2_FC) for each identified protein in replicate 1 vs. replicate 2. The Pearson correlation between the two replicates is 0.751 (*p* < 0.0001). **(D)** Analysis of differentially immunoprecipitated proteins between Mock and co-IP conditions. The plot shows the Log_2_FC vs. the total peptide abundance; where the *Average.Top3* is the average intensity for the three most abundant peptides of an individual protein and the denominator is the sum of all Top3 values in a condition. Proteins with a Log_2_FC ≥2 and an Average.Top3 ≥7 represents the optimal interacting proteins, which were used for further analysis. Proteins highlighted with cream dots have already been functionally related to M6a. Proteins highlighted with green dots and blue dots were selected for ulterior proteomic validation.

**Figure 3:**
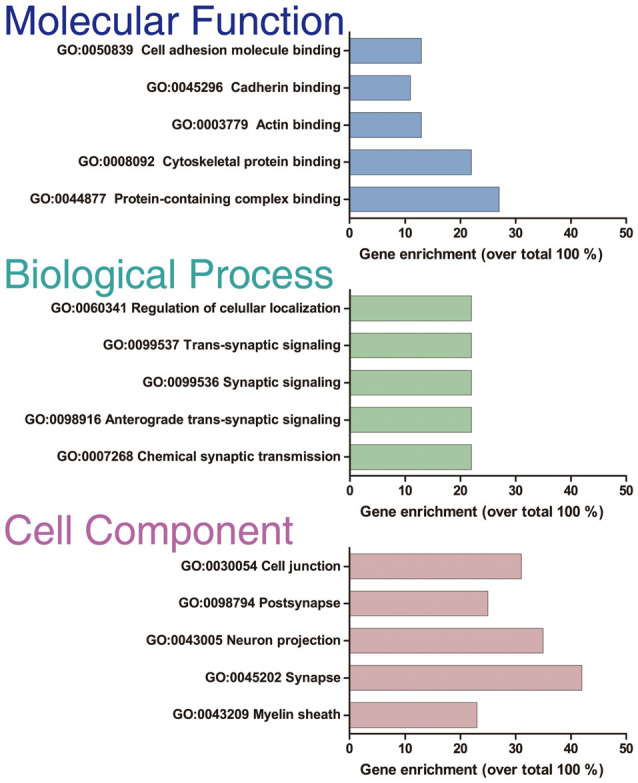
GO enrichment analysis. GO enrichment analysis of the 414 optimal interacting proteins. The analysis was done with the ToppFun application of the ToppGene Suit server. The plot shows the top five categories in which the proteins were classified for each category analyzed: “Molecular function,” “Biological process,” and “Cellular component.”

Only proteins that were quantified with at least two unique peptide matches were kept for the analysis. Moreover, proteins were kept only if they were quantified in at least 2/3 of the independent experiments. In Supplementary Table S1 we summarize the total 1,529 identified proteins that passed these quality filtration steps, with their corresponding maximum number of peptides recognized in one run. The raw signal data obtained (Figure 2A) were subjected to batch effect removal, data transformation, and logarithmic fold changes (log_2_FC) calculation for ulterior comparison (Figure 2B). From the quantitative proteomic data, we performed Limma analysis which allowed us to identify differentially immunoprecipitated peptides/proteins between conditions. The Pearson correlation coefficient between log2FC of biological replicates was r 0.751 (*p* < 0.0001; Figure 2C). Therefore, we averaged the two replicates within each independent co-IP assay and plotted the log_2_FC against the log of the total peptide intensity (average.top3), which can be used as a proxy for absolute protein abundance (Figure 2D and Supplementary Table S2). The highest-ranked proteins, proteins whose average of the relative abundance of their three best peptides (average.top3) was greater than 7 and with a log_2_FC of at least two were kept for further analysis (Werner et al., 2014; Franken et al., 2015; Ritchie et al., 2015; Jacquemet et al., 2019). Interestingly, we also identified proteins that were functionally associated with M6a in previous reports. For example, protein RUFY3 and neural cell adhesion molecule 1 (NCAM1) are linked to M6a at the early stage of neuron development (Sato et al., 2011). Whereas glutamate ionotropic receptor, GRIN1 (also called NMDA-R1) and synaptophysin (SYP) were functionally associated with M6a at the synaptic cleft in a mature culture of hippocampal neurons (Alfonso et al., 2005; Formoso et al., 2016; Garcia et al., 2017; cream dots in Figure 2D). Hereafter, we characterized the global protein pattern using the list of 414 genes (Supplementary Table S2) as an input for the ToppGene Suite server^2^. Using the ToppFun tool we performed GO and enrichment analysis to prioritize statistically significant genes (Chen et al., 2009). The top five GO function categories of these proteins are shown in Figure 3 and Supplementary Table S3. Regarding the biological processes category, enrichment analysis showed that the most significant enrichment was for proteins involved in “synaptic transmission” and “synaptic signaling.” This correlates with the GO enrichment of the “cellular component” category in which most proteins were significantly identified in “synapse,” “cell junction,” “postsynapse,” “neuron projection” and “myelin sheath” compartments. In the case of the “molecular function” category, the most significant terms displayed are related to binding proteins.

**Figure 4:**
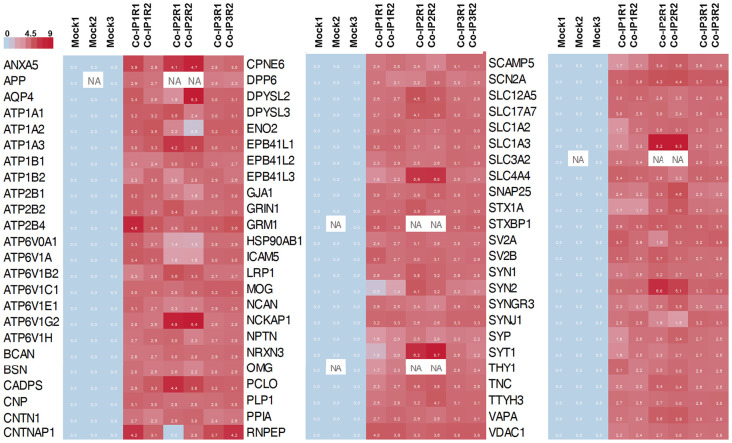
Heat map representation of the 72 differentially abundance proteins. The heat map shows the abundance of each protein in each co-IP (and replicates) compare to the control condition (Mock). In the color scale, sky blue represents proteins with low levels, and red represents proteins with high levels of abundance. White spots represent proteins with no signal available (NA).

Subsequently, we manually curated the 414 listed proteins based on literature and subcellular localization annotation according to the UniProt database^1^. We excluded proteins localized in nucleus, cytosol or organelles (Golgi apparatus, mitochondria, and endoplasmic reticulum), whereas we kept proteins placed in the plasma membrane, extracellular matrix, synaptic vesicles membranes or secreted ones (Végh et al., 2014; Jacquemet et al., 2019; Lleó et al., 2019). Thus, the remaining list of 72 proteins, due to their subcellular localization, might interact with the extracellular loops of M6a. Figure 4 shows a heatmap of the abundance ratio of each protein in each co-IP compared to their corresponding Mock condition. The heatmap color scale is shown in the left upper corner, with red being high and sky blue being low levels. The white square represents samples with no signal available (NA). We performed a new GO enrichment analysis with the 72 M6a’s potential interactors using the ToppFun tool and the results are shown in Table 1 (details in Supplementary Table S4). Again, the top five GO annotations are significantly related to synaptic transmission/signaling associated with proteins, mainly ion transporters, on both sides of the synaptic cleft. Using the same approach we also identified and prioritized novel disease candidate genes within the 72 proteins by ToppGene Suite server throughout a DisGeNET database^4^. The results are summarized in Table 2. Interestingly, in the top 10 diseases “Schizophrenia,” “Alzheimer’s Disease,” “Depressive Disorder, “Major Depressive Disorder,” “Bipolar Disorders,” and “Mental Depression” were found, which have already been associated with glycoprotein M6a (Boks et al., 2008; Greenwood et al., 2012; El-Kordi et al., 2013; Gregor et al., 2014; Fuchsova et al., 2015; Lachén-Montes et al., 2016; Jacquemet et al., 2019).

**Table 1:** Gene ontology (GO) enrichment analysis of 72 potential M6a’s interactors.

	ID	Annotation	*p*-value	FDR B&H	FDR B&Y	Bonferroni	Genes from input (72)	Protein symbol
Molecular function	GO:0022853	active ion transmembrane transporter activity	1.562E-20	3.85E-18	2.602E-17	7.545E-18	22 (31%)	Atp6v1a, Atp6v1b2, Atp6v1c1, Atp6v1e1, Atp6v1g2, Atp6v1h, Atp6v0a1, Anxa5, Slc17a7, Atp1a1, Atp1a2, Atp1a3, Slc4a4, Atp1b1, Atp1b2, Atp2b1, Slc1a2, Atp2b2, Slc1a3, Atp2b4, Slc3a2, Slc12a5
	GO:0005215	transporter activity	1.631E-20	3.85E-18	2.602E-17	7.878E-18	38 (53%)	Gja1, Atp6v1a, Dpp6, Atp6v1b2, Atp6v1c1, Atp6v1e1, Cpne6, Stx1a, Atp6v1g2, Atp6v1h, Atp6v0a1, Nrxn3, Sv2b, Sv2a, Anxa5, Scn2a, Syngr3, Syn1, Slc17a7, Grin1, Ttyh3, Snap25, Atp1a1, Atp1a2, Atp1a3, App, Slc4a4, Atp1b1, Atp1b2, Aqp4, Atp2b1, Slc1a2, Atp2b2, Slc1a3, Atp2b4, Slc1a3, Atp2b4, Slc3a2, Vdac1, Slc12a5
	GO:0019829	ATPase-coupled cation transmembrane transporter activity	2.391E-20	3.85E-18	2.602E-17	1.155E-17	16 (22%)	Atp6v1a, Atp6v1b2, Atp6v1c1, Atp6v1e1, Atp6v1g2, Atp6v1h, Atp6v0a1, Anxa5, Atp1a1, Atp1a2, Atp1a3, Atp1b1, Atp1b2, Atp2b1, Atp2b2, Atp2b4
	GO:0042625	ATPase-coupled ion transmembrane transporter activity	5.2E-20	6.279E-18	4.244E-17	2.512E-17	16 (22%)	Atp6v1a, Atp6v1b2, Atp6v1c1, Atp6v1e1, Atp6v1g2, Atp6v1h, Atp6v0a1, Anxa5, Atp1a1, Atp1a2, Atp1a3, Atp1b1, Atp1b2, Atp2b1, Atp2b2, Atp2b4
	GO:0022857	transmembrane transporter activity	7.159E-19	6.916E-17	4.674E-16	3.458E-16	35 (49%)	Gja1, Atp6v1a, Dpp6, Atp6v1b2 Atp6v1c1, Atp6v1e1, Stx1a, Atp6v1g2, Atp6v1h, Atp6v0a1, Nrxn3, Sv2b, Sv2a, Anxa5, Scn2a, Slc17a7, Grin1, Ttyh3, Snap25, Atp1a1, Atp1a2, Atp1a3, App, Slc4a4, Atp1b1, Atp1b2, Aqp4, Atp2b1, Slc1a2, Atp2b2, Slc1a3, Atp2b4, Slc3a2, Vdac1, Slc12a5
Biological Process	GO:0098916	anterograde trans-synaptic signaling	2.463E-20	2.819E-17	2.373E-16	6.274E-17	31 (43%)	Dpp6, Nptn, Cpne6, Stx1a, Nrxn3, Stxbp1, Synj1, Cadps, Sv2b, Sv2a, Pclo, Scn2a, Cntnap1, Syn1, Slc17a7, Syn2, Syp, Syt1, Grin1, Snap25, Atp1a2, App, Bsn, Grm1, Plp1, Slc1a2, Atp2b2, Slc1a3, Cnp, Vdac1, Slc12a5
	GO:0007268	chemical synaptic transmission	2.463E-20	2.819E-17	2.373E-16	6.274E-17	31 (43%)	Dpp6, Nptn, Cpne6, Stx1a, Nrxn3, Stxbp1, Synj1, Cadps, Sv2b, Sv2a, Pclo, Scn2a, Cntnap1, Syn1, Slc17a7, Syn2, Syp, Syt1, Grin1, Snap25, Atp1a2, App, Bsn, Grm1, Plp1, Slc1a2, Atp2b2, Slc1a3, Cnp, Vdac1, Slc12a5
	GO:0099537	trans-synaptic signaling	3.319E-20	2.819E-17	2.373E-16	8.456E-17	31 (43%)	Dpp6, Nptn, Cpne6, Stx1a, Nrxn3, Stxbp1, Synj1, Cadps, Sv2b, Sv2a, Pclo, Scn2a, Cntnap1, Syn1, Slc17a7, Syn2, Syp, Syt1, Grin1, Snap25, Atp1a2, App, Bsn, Grm1, Plp1, Slc1a2, Atp2b2, Slc1a3, Cnp, Vdac1, Slc12a5
	GO:0099536	synaptic signaling	5.078E-20	3.235E-17	2.724E-16	1.294E-16	31 (43%)	Dpp6, Nptn, Cpne6, Stx1a, Nrxn3, Stxbp1, Synj1, Cadps, Sv2b, Sv2a, Pclo, Scn2a, Cntnap1, Syn1, Slc17a7, Syn2, Syp, Syt1, Grin1, Snap25, Atp1a2, App, Bsn, Grm1, Plp1, Slc1a2, Atp2b2, Slc1a3, Cnp, Vdac1, Slc12a5
	GO:0030001	metal ion transport	1.414E-18	7.204E-16	6.067E-15	3.602E-15	31 (43%)	Gja1, Atp6v1a, Dpp6, Atp6v1b2, Atp6v1c1, Atp6v1e1, Stx1a, Atp6v1g2, Atp6v1h, Atp6v0a1, Thy1, Anxa5, Scn2a, Slc17a7, Grin1, Snap25, Atp1a1, Atp1a2, Atp1a3, Slc4a4, Atp1b1, Atp1b2, Atp2b1, Slc1a2, Atp2b2, Slc1a3, Atp2b4, Epb41, Cntn1, Slc3a2, Vdac1, Slc12a5
Celular Component	GO:0045202	synapse	1.406E-30	5.552E-28	3.641E-27	5.552E-28	45 (63%)	Nptn, Dpysl2, Dpysl3, Stx1a, Atp6v1g2, Atp6v0a1, Nrxn3, Stxbp1, Nckap1, Synj1, Scamp5, Cadps, Sv2b, Sv2a, Anxa5, Pclo, Scn2a, Ncan, Syngr3, Cntnap1, Syn1, Slc17a7, Syn2, Syp, Syt1, Bcan, Grin1, Snap25, Atp1a1, Atp1a2, Atp1a3, App, Bsn, Grm1, Epb41l3, Atp2b1, Eno2, Slc1a2, Atp2b2, Slc1a3, Atp2b4, Epb41, Cntn1, Slc3a2, Vdac1
	GO:0098793	presynapse	2.191E-29	4.328E-27	2.838E-26	8.656E-27	35 (49%)	Nptn, Dpysl2, Stx1a, Atp6v1g2, Atp6v0a1, Nrxn3, Stxbp1, Synj1, Scamp5, Cadps, Sv2b, Sv2a, Anxa5, Pclo, Scn2a, Syngr3, Cntnap1, Syn1, Slc17a7, Syn2, Syp, Syt1, Grin1, Snap25, Atp1a2, Atp1a3, App, Bsn, Grm1, Atp2b1, Slc1a2, Atp2b2, Atp2b4, Cntn1, Vdac1
	GO:0043209	myelin sheath	1.714E-20	2.257E-18	1.48E-17	6.772E-18	21 (28%)	Atp6v1a, Atp6v1b2, Dpysl2, Stxbp1, Thy1, Cntnap1, Syn1, Syn2, Snap25, Atp1a1, Atp1a3, Atp1b1, Ppia, Eno2, Plp1, Omg, Cnp, Mog, Cntn1, Hsp90, Vdac1
	GO:0030054	cell junction	1.073E-19	1.052E-17	6.89E-17	4.208E-17	35 (63%)	Vapa, Gja1, Stx1a, Thy1, Nckap1, Scamp5, Cadps, Sv2b, Tnc, Sv2a, Anxa5, Pclo, Scn2a, Syngr3, Cntnap1, Lrp1,
								Syn1, Slc17a7, Syn2, Syp, Syt1, Grin1, Snap25, Atp1a1, Atp1a2, App, Bsn, Epb41l3, Atp1b1, Ppia, Aqp4, Atp2b1, Atp2b2, Epb41, Epb41l2
	GO:0043005	neuron projection	2.962E-19	2.925E-17	1.918E-16	1.17E-16	37 (51%)	Nptn, Dpysl2, Dpysl3, Cpne6, Stx1a, Stxbp1, Thy1, Rnpep, Synj1, Sv2b, Sv2a, Anxa5, Pclo, Scn2a, Cntnap1, Lrp1, Syn1, Slc17a7, Syp, Syt1, Bcan, Grin1, Snap25, Atp1a2, Atp1a3, App, Bsn, Grm1, Epb41l3, Atp2b1, Eno2, Slc1a2, Atp2b2, Slc1a3, Atp2b4, Hsp90, Slc12a5

*The table shows the top five GO classification for each category analyzed: “Molecular function,” “Biological process” and “Cellular component.” The analysis was done with the ToppFun application of the ToppGene Suit server*.

**Table 2:** Disease-associated gene analysis of the 72 potential M6a’s interactors.

	ID	Annotation	*p*-value	FDR B&H	FDR B&Y	Bonferroni	Genes from input (72)	Protein symbol
Disease	C0004352	Autistic Disorder	1.84E-10	3.041E-07	2.43E-06	3.041E-07	20 (27%)	Gja1, Dpysl2, Stx1a, Nrxn3, Stxbp1, Thy1, Scamp5, Tnc, Pclo, Scn2a, Cntnap1, Syn1, Syn2, Snap25, App, Aqp4, Plp1, Slc1a2, Atp2b2, Slc1a3
	C0014544	Epilepsy	1.05E-09	8.625E-07	6.89E-06	1.73E-06	19 (26%)	Gja1, Dpysl3, Stx1a, Stxbp1, Sv2a, Scn2a, Syn1, Slc17a7, Syn2, Grin1, Atp1a2, Atp1a3, App, Grm1, Aqp4, Slc1a2, Slc1a3, Vdac1, Slc12a5
	C0036341	Schizophrenia	2.40E-09	1.32E-06	1.05E-05	3.96E-06	28 (38%)	Nptn, Dpysl2, Stx1a, Nrxn3, Stxbp1, Sv2a, Pclo, Scn2a, Ncan, Lrp1, Syn1, Slc17a7, Syn2, Syp, Bcan, Grin1, Snap25, App, Grm1, Ppia, Aqp4, Eno2, Plp1, Slc1a2, Slc1a3, Cnp, Mog, Slc12a5
	C0002395	Alzheimer’s Disease	2.10E-05	6.93E-03	0.055	0.035	26 (34%)	Gja1, Dpysl2, Dpysl3, Thy1, Synj1, Sv2a, Anxa5, Lrp1, Syn1, Slc17a7, Syn2, Syp, Syt1, Bcan, Grin1, Snap25, App, Ppia, Aqp4, Eno2, Slc1a2, Slc1a3, Cnp, Mog, Hsp90, Vdac1
	C0086237	Epilepsy, Cryptogenic	5.30E-06	2.19E-03	0.017	0.009	7 (9, 7%)	Gja1, Scn2a, Grm1, Slc1a2, Slc1a3, Vdac1, Slc12a5
	C0005586	Bipolar Disorder	2.61E-05	7.18E-03	0.057	0.043	16 (22%)	Vapa, Dpysl2, Synj1, Pclo, Ncan, Slc17a7, Syn2, Syp, Grin1, Snap25, Atp1a1, Atp1a2, Atp1a3, App, Slc1a2, Slc1a3
	C0036572	Seizures	4.81E-05	0.010	0.081	0.079	14 (19%)	Gja1, Stxbp1, Synj1, Sv2a, Scn2a, Slc17a7, Syn2, Grin1, Atp1a2, App, Aqp4, Slc1a2, Slc1a3, Slc12a5
	C0011570	Mental Depression	4.92E-05	0.010	0.081	0.081	14 (19%)	Gja1, Dpysl2, Pclo, Syn1, Syp, Grin1, Snap25, Atp1a3, App, Grm1, Aqp4, Slc1a2, Cnp, Hsp90
	C1269683	Major Depressive Disorder	1.27E-04	2.11E-02	1.70E-01	2.19E-01	13 (19%)	Gja1, Pclo, Ncan, Lrp1, Slc17a7, Snap25, App, Plp1, Slc1a2, Slc1a3, Cnp, Mog, Hsp90
	C0011581	Depressive disorder	1.28E-04	0.022	0.176	0.211	14 (19%)	Gja1, Dpysl2, Pclo, Syn1, Syp, Grin1, Snap25, Atp1a3, App, Grm1, Aqp4, Slc1a2, Cnp, Hsp90

*The table shows the overlap of the associated genes in the top 10 diseases. The analysis was done with the ToppFun application of the ToppGene Suit using data sourced from DisGeNET*.

### Confirmation by Immunostaining

Based on their subcellular localization and being part of the most represented cellular-component observed in the enrichment analysis, four proteins were selected to confirm whether or not they may interact with M6a (shown as green dots in Figure 2D). Forty-nine percent of the proteins are located in the synapse, with the majority of them (35/45) being pre-synaptic proteins. Thus, we selected one protein from the synaptic vesicle membrane (SV2B), one from the cytomatrix (PCLO) and one that coats synaptic vesicles (SYN1). In third place, 28% of the proteins were assigned to the “myelin sheath” cellular compartment, so we also selected the oligodendrocyte cell surface protein PLP for colocalization assays. In previous work, we determined that M6a and transferrin receptor, TfR or TFRC, share the same clathrin dependent-endocytic pathway (Garcia et al., 2017). Nonetheless, TFRC was excluded from subsequent analyses due to low relative abundance (average.top.3) of 6,6 and a log2FC of 2.05. Thus, TfR was considered as a negative control in the colocalization assays (TFRC, the blue dot in Figure 2D).

#### M6a Interacts With Synaptic Proteins

**Figure 5:**
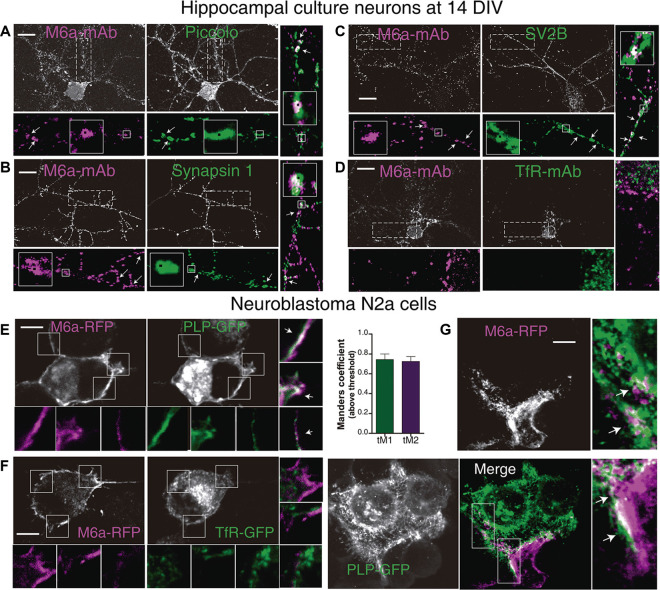
M6a interacts with synaptic proteins and with major myelin protein, PLP. **(A–D)** Representative images of primary hippocampal cultured neurons at 14 DIV. Neurons were labeled for endogenous M6a (gray or magenta in the insets) and endogenous piccolo **(A)**, synapsin 1 **(B)**, SV2B **(C)**, and TfR (**(D**; gray and green in the insets). The magnifications show 25 μm of primary **(C,D)**, secondary **(A)**, and tertiary **(A,B)** dendrite length. Punta Analyzer plugin of ImageJ was used to measure the colocalization between M6a and synaptic markers. Puncta Analyzer displayed images such as the ones shown here in which colocalization puncta are indicated by black squares (inset). Merged images show clusters of M6a colocalizing with clusters of piccolo **(A)** or synapsin-1 **(B)** and SV2B (**C**; with arrows and black squares). Scale bar: 10 μm. **(E,F)** Representative images of murine neuroblastoma N2a cells co-transfected with M6a-RFP/PLP-GFP **(E)** and M6a-RFP/TfR-GFP **(F)**. The insets (5 × 5 μm) show the pattern of distribution of M6a-RFP (magenta) and PLP-GFP or TfR-GFP (green) and the merged images show colocalization between M6a-RFP and PLP-GFP (**E**, white arrows), but not between M6a-RFP and TfR-GFP. **(G)** Representative images of co-culture experiment between cells that only expressed PLP-GFP and cells that only expressed M6a-RFP. The magnification (5 × 10 μm) reveals patches of colocalization (white) between both cell surfaces. Scale bar: 5 μm. Confocal images were acquired with a 60× objective on an Olympus FV1000 confocal microscope. The colocalization between M6a and TfR or PLP was measured by the Coloc2 plugin of ImageJ. For those pairs of proteins positive for colocalization (average of Pearson’s coefficient over than 0.5). Mander’s coefficients (M1, green channel and M2, red channel) were measured and plotted (*n* = 13–25 cells). No positive Pearson’s coefficients of colocalization between endogenous M6a and TfR or M6a-RFP and TfR-GFP were measured.

Axon terminals—pre-synaptic compartment—form multiple synaptic contacts with the cell soma and dendrites—post-synaptic compartment—of another neuron aided by adhesion molecules that interact across the synaptic cleft (Scheiffele, 2003; Sytnyk et al., 2004; Biederer and Scheiffele, 2007). These specialized junctions between neurons can be easily identified and quantified as the number of colocalization of immunoreactive synaptic clustered proteins along dendrites in cultured neurons (Dzyubenko et al., 2016; Verstraelen et al., 2018). Thus, we examined whether M6a might associate with the selected synaptic proteins by immunostaining primary hippocampal neurons at 12–15 DIV with the appropriate antibodies. Piccolo, synapsin 1 and the synaptic vesicle glycoprotein 2B-SV2B-exhibit a normal pre-synaptic polarity, meaning these proteins become preferentially accumulated, in a punctate manner, in axon terminals (Fletcher et al., 1991; Leal-Ortiz et al., 2008; Terry-Lorenzo et al., 2016; Bartholome et al., 2017). Regarding M6a, we previously determined that it is distributed along the neuronal surface within both pre- and post-synaptic compartments as discrete puncta (Formoso et al., 2016). Therefore, we first selected microscopic fields in which pre-synaptic positive axon terminals were contacting the secondary/tertiary dendrite of another neuron. Second, we quantified the puncta colocalization between M6a puncta and PCLO or SYN1 or SV2B puncta. Considering the punctate distribution of the synaptic proteins, the Puncta Analyzer plugin of ImageJ—which measures colocalization by an object-based method—was used (Ippolito and Eroglu, 2010; Dunn et al., 2011). Figures 5A–C shows a representative image of each condition and the output obtained from the Puncta Analyzer plugin as black squares (arrows and magnified insets). Figure 5A shows a representative image of a 14 DIV hippocampal neuron and its magnification (25 μm of secondary and tertiary dendrites length), where we observed dots of colocalization (white with a black square) between M6a puncta (magenta) and piccolo puncta (green). Figure 5B shows a representative image of tertiary dendrite and its magnification in which we observed dots of colocalization (white with black squares) between clusters of endogenous M6a (magenta with black squares) with clusters of synapsin 1 (green with black squares). Here again, the white color of overlapping between M6a puncta (magenta with black squares) and SV2B puncta (green with black squares) was also observed along the secondary dendrite in the representative hippocampal neuron in Figure 5C (for more details see Supplementary Figure S3). By contrast, no colocalization pattern was observed between M6a (magenta) and endogenous transferrin receptor (green) at the soma and along the dendrites of the neuron (Figure 5D). Considering the TFRC staining distribution, we quantified the possible colocalization by an intensity-based method using the Coloc2 plugin of ImageJ (Bolte and Cordelieres, 2006). At least three regions of interest per cell were defined in the cell surface and analyzed. The average of Pearson’s coefficient between both proteins was −0.238 ± 0.039 (*n* = 15 cells) supporting previous observation. Taken together, as retrieved by co-IP-TMT/MS followed by bioinformatics tools we corroborated the colocalization between M6a and the pre-synaptic markers assessed.

#### M6a Interacts With the Major Myelin Protein—PLP

Proteolipid protein, PLP, is the most abundant integral membrane protein of the CNS and expresses mainly in oligodendrocytes (Lüders et al., 2019). We further investigated whether M6a associates with PLP in the plasma membrane of co-transfected neuroblastoma N2a cells. Thus, N2a cells were transiently co-transfected with M6a tagged with RFP (M6a-RFP)/PLP tagged with GFP (PLP-GFP) or M6a-RFP/TfR-GFP. As it is shown in Figure 4E, PLP (green) is an integral component of the cell surface, however, it is also localized in the lysosome as storage compartment (Winterstein et al., 2008). M6a (magenta) overexpressing N2a cells showed a similar distribution that was described in neurons. A colocalization pattern (white arrows in the insets of 5 × 5 μm), like patches, all along the cell membrane, and cell protrusions were observed between both proteins. Conversely, no color superposition was observed in the cell surface and processes of M6a/TfR-coexpressing N2a cells (Figure 5F). Taking into consideration that the most common scenario is that M6a and PLP associate in *trans*, which means cell-cell (neuron-glia) interaction, we designed a co-culture experiment. Thus, two pools of cells separately transfected with PLP-GFP and M6a-RFP were used. Transfected pool cells were then mixed and allowed to interact for 3 h and then were subjected to immunofluorescence. Figure 5G reveals patches of colocalization (white in the magnification) between both cell surface protrusions. Here again, a quantitative estimation of colocalization was performed using the Coloc2 plugin. Pearson coefficient’s between M6a and PLP was 0.520 ± 0.047 (*n* = 13 cells) and between M6a and TfR was 0.048 ± 0.032 (*n* = 25 cells). The Mander’s coefficients above the threshold between M6a (red channel, M2) and PLP (green channel, M1) were plotted in Figure 5F showing a high level of overlap between both fluorescence signals. In summary, as in the case of synaptic markers, the results support the association between M6a and PLP.

## Discussion

The cellular and molecular mechanisms governing the formation of synapses and developmental plasticity, including the roles of cell-cell recognition molecules, cell adhesion molecules, growth factors, electrical activity among others are critical for the normal brain development. The relationship between cells and their surroundings is markedly mediated by proteins expressed on the cell surface. Despite the functional role of the M6a’s extracellular loops, there is still no evidence about physiological partners. In this work, we used a co-IP-TMT/MS approach followed by bioinformatics analysis, and we identified 72 potential M6a’s interactors of which four proteins were confirmed by colocalization.

We recently reported that the extracellular loops of M6a and cysteine residues within the EC2 play a critical role for M6a folding, trafficking and neuronal plasticity (Lagenaur et al., 1992; Fuchsova et al., 2009; Formoso et al., 2016; Garcia et al., 2017). In this sense, tetraspanins are a large family of proteins in which the EC2 is a critical domain involved in the specific intermolecular interactions (van Deventer et al., 2017). Moreover, the high-resolution cryoelectron microscopy structure of the tetraspanin protein family showed that the EC1 domain packs in under the EC2 domain (extracellular primary interaction). After, the EC1-EC2 binds to the extracellular region of the partner protein (secondary interaction) across the hypervariable region of EC2 (Min et al., 2006). Likewise, proteolipid protein family (PLP1/DM20, M6a, and M6b) structure are somewhat similar to tetraspanins, and, therefore, a few studies have characterized the specific binding sequences of PLPs to their partners, arguing that their EC2 acts as a determinant site (Lagenaur et al., 1992; Dhaunchak and Nave, 2007; Winterstein et al., 2008; Dhaunchak et al., 2011; Sato et al., 2011; Formoso et al., 2015a). In this work, to discover M6a ligands/partners we successfully cloned, expressed and characterized a chimera protein that contained both M6a’s extracellular loops (M6a-loops). A similar approach, immunoprecipitation followed by mass spectrometry, has been used to describe the association of tetraspanin CD9 with the major histocompatibility complex MHC class II molecule, α6b1 integrin, and CD44 in the cell surface of B-lymphoid and platelets cells (Le Naour et al., 2004). Regarding M6a (Honda et al., 2017), using co-IP followed by mass spectrometry identified nine proteins (GPM6A, GPM6B, Sez6l2, Lyric, TMEM30A, Xpr1, Tpbgl, Creld1, and Fndc3a) from FLAG-tagged full M6a overexpressing HEK293 cells. Apart from GPM6A (here, *6,9/3,0* average.top3/log_2_FC, Supplementary Table S2), none of them were confirmed by our approach. Nevertheless, the oligomerization of M6a in cell surface from rat hippocampi and cultured neurons has been already proven (Formoso et al., 2015b). However, when (Honda et al., 2017) co-immunoprecipitated Triton X-100 extracts from mice neuronal growth cone membrane fractions using M6a-mAb; they identified and confirmed by immunostaining the endogenous association between M6a and Rufy3. Despite having identified Rufy3 (*3,07/7,3*; Figure 2D), it was cut off from the analysis because of its subcellular localization, which would suggest a direct interaction with the M6a cytoplasmic tails instead of its extracellular domains. We speculate that M6a-loops interacted, under non-reducing conditions, with endogenous M6a (see Supplementary Figure S2) and altogether co-precipitated with its C- and N-tails partners. This could also explain why clathrin, coronin 1A, HSP70, Src kinase family, phosphatidylinositide 3-kinase, Rab-5, Rab-7, Rab-11, Rac-1/PaK, HIP14, PSMC5, and PKC were identified (Supplementary Tables S1, S2), which have been associated with M6a signal transduction, traffic and function (Mukobata et al., 2002; Sato et al., 2011; Scorticati et al., 2011; Butland et al., 2014; Formoso et al., 2015a; Fuchsova et al., 2015; Alvarez Juliá et al., 2016; Garcia et al., 2017; Ramachandran and Margolis, 2017).

In previous work we demonstrated that M6a plays a role in synapse formation and maintenance; increasing the number of clusters of the pre-synaptic component synaptophysin (*SYP*) and the post-synaptic component glutamate receptor ionotropic NMDA-R1 (*GRIN1*, Formoso et al., 2016). Notably, 45 of the curated proteins, approximately 63% of total, were classified by their cellular component as “synapse” (GO:0045202, Table 1) in which 35 were “presynapse” (GO:0098793: Nptn, Dpysl2, Stx1a, Atp6v1g2, Atp6v0a1, Nrxn3, Stxbp1, Synj1, Scamp5, Cadps, Sv2b, Sv2a, Anxa5, Pclo, Scn2a, Syngr3, Cntnap1, Syn1, Slc17a7, Syn2, Syp, Syt1, Grin1, Snap25, Atp1a2, Atp1a3, App, Bsn, Grm1, Atp2b1, Slc1a2, Atp2b2, Atp2b4, Cntn1, Vdac1) and 37 were part of “neuron projection” (GO:0043005: Nptn, Dpysl2, Dpysl3, Cpne6, Stx1a, Stxbp1, Thy1, Rnpep, Synj1, Sv2b, Sv2a, Anxa5, Pclo, Scn2a, Cntnap1, Lrp1, Syn1, Slc17a7, Syp, Syt1, Bcan, Grin1, Snap25, Atp1a2, Atp1a3, App, Bsn, Grm1, Epb41l3, Atp2b1, Eno2, Slc1a2, Atp2b2, Slc1a3, Atp2b4, Hsp90, Slc12a5). Here, we confirmed by immunostaining the association between endogenous M6a and piccolo (*PCLO 2,7/*7,9; Figure 2D), synaptic vesicle protein 2 B (*SV2B* *3,0/7,5*; Figure 2D) and synapsin 1 (*SYN1*, *2,7/8,2*; Figure 2D) in rat neuronal cultures of 14 DIV (Figures 5A–D). M6a has been isolated from membranes of synaptic vesicles of the adult rat brain (Takamori et al., 2006). Thus, M6a located at the surface of the synaptic vesicle might interact with SV2B in *cis* in the same vesicle or *trans* with a vesicle in its neighborhood. In the same context, M6a might interact with the scaffold proteins of the presynaptic cytomatrix at the active zone, piccolo, and synapsin 1.

Although the interaction between M6a with pre-synaptic receptors such as the μ-opioid receptor, cannabinoid receptor CB1, and somatostatin receptor sst2A was documented, they were ruled out from our study (Wu et al., 2007). Moreover, in the same work, the association between M6a and the metabotropic glutamate receptor 1 (here GRM1, *3,1/7,0*; Figure 2D) was excluded by bioluminescence resonance energy transfer (BRET) assay in cells transiently transfected with both proteins. This could be explained because the authors use a truncated form of M6a (108-278 M6a) lacking N-tail, TMD1, and EC1- interactions from their assays.

Among others, 21 of the selected proteins (28%) were annotated as part of the “myelin sheath” (GO:0043209: Atp6v1a, Atp6v1b2, Dpysl2, Stxbp1, Thy1, Cntnap1, Syn1, Syn2, Snap25, Atp1a1, Atp1a3, Atp1b1, Ppia, Eno2, Plp1, Omg, Cnp, Mog, Cntn1, Hsp90, Vdac1). Therefore, M6a may interact with membrane proteins expressed on the surface of CNS oligodendrocytes. By immunostaining, we confirmed the association between M6a and PLP in co-expressing N2a cells (*cis* interaction) and co-culture experiment (*trans* interaction; Figures 5E–G). Remarkably, M6b was identified as a Schwann cell microvilli component that stabilizes the nodes of Ranvier by its association with glial gliomedin, CNTNAP1 (here 2,6/7,8), glial NrCAM (here 2,1/8,0), and axonal NF186 (here 3,5/6,8) in a co-culture of dorsal root ganglion neurons and Schwann cells (Bang et al., 2018). Although, there is no evidence supporting the role of M6a in the peripheral nervous system; our data suggest that M6a could participate as a regulator of sensory neuron and glia interactions.

In this work, with an integrative platform (DisGeNET) by which human-disease associated genes and variants are classified, the final 72 proteins were analyzed (Piñero et al., 2017). Not surprisingly, the analysis displayed the names of disorders that have been already linked to *GPM6A* altered expression or *GPM6A* genetic variants in human or animal models of diseases (Alfonso et al., 2002, 2004, 2005; Greenwood et al., 2012; El-Kordi et al., 2013; Penzes et al., 2013; Gregor et al., 2014; Formoso et al., 2015b; Fuchsova et al., 2015; Mita et al., 2015; Lachén-Montes et al., 2016). Moreover, new terms such as “autistic disorder,” “epilepsy” and, “seizures” were found, suggesting a new role of M6a in these disorders.

In summary, the main aim of this project was to augment the comprehension of the molecular mechanisms linked to neuronal plasticity and thus improve the assessment, diagnosis, and treatment of many nervous system disorders among others. Fluorescence immunocytochemistry followed by quantification of protein colocalization is an indirect technique for determining protein-protein interaction. Hence, experiments like *in situ* proximity ligation assay, PLA (Alam, 2018; Almandoz-Gil et al., 2018), which confirm the direct interaction between two proteins are needed for the final validation of our study. Besides, experiments focused on the functional implication of the potential M6a’s interactors are convenient to understand the integral role of M6a in the neuronal development and the neurobiology of the diseases.

## Data Availability Statement

The mass spectrometry proteomics data have been deposited to the ProteomeXchange Consortium (www.proteomexchange.org) *via* the PRIDE (Vizcaíno et al., 2016) partner repository with the dataset identifier PXD017347.

## Ethics Statement

The animal study was reviewed and approved by the Committee for the Care and Use of Laboratory Animals of the Universidad Nacional de San Martín (CICUAE-UNSAM), CICUAE-UNSAM No. 03/2015 and CICUAE-UNSAM No. 03/2016.

## Author Contributions

GA, KF, AL, AF, and CS were involved in the design of experiments and analysis of data; mainly GA performed experiments with the help of KF and AL. CS, GA, and AL wrote the manuscript. KF and AF critically revised the manuscript.

## Conflict of Interest

The authors declare that the research was conducted in the absence of any commercial or financial relationships that could be construed as a potential conflict of interest.

## Footnotes

^1^https://www.uniprot.org/

^2^https://toppgene.cchmc.org/

^3^https://www.r-project.org

^4^http://www.disgenet.org/

## Abbreviations

BAP: Biotin Acceptor Peptide
CE: Cellular extract
CH3: Human IgG heavy chain constant domain 3
co-IP: Co-immunoprecipitation
DB: Dot blot
DDA: Data Dependent Acquisition
DIV: Days *in vitro*
EC1: Extracellular domain 1 or small extracellular loop
EC2: Extracellular domain 2 or large extracellular loop
FC: Fold change
FDR: False Discovery Rate
GO: Gene Ontology
GRIN1: N-methyl-D-aspartate receptor type 1
HEK293: Human Embryonic Kidney cells
HP: Pooled hippocampi
Limma: Linear Models for Microarray Data
MS: Mass spectrometry
N2a: Murine neuroblastoma N2a cells
NCAM: Neuronal Cell Adhesion Molecule
PCLO: Piccolo
PLP1: Proteolipid Protein 1
PKC: Protein Kinase C
P: Postnatal day
SEC: Secretion sequence
SN: Supernatant
SV2B: Synaptic vesicle protein 2B
SYN1: Synapsin 1 protein
TMT: Tandem Mass Tag
TMT/MS: Tandem mass tag spectrometry identification
TRFC: Transferrin receptor
WB: Western blot.
