# Supplementary material for: Identification of Potential Interacting Proteins With the Extracellular Loops of the Neuronal Glycoprotein M6a by TMT/MS
Source: Front Synaptic Neurosci. 2020 Jul 23;12:28. doi: 10.3389/fnsyn.2020.00028 (PMC7396582; doi:10.3389/fnsyn.2020.00028)
Supplement: Supplementary file 1 [file Data_Sheet_1.PDF]

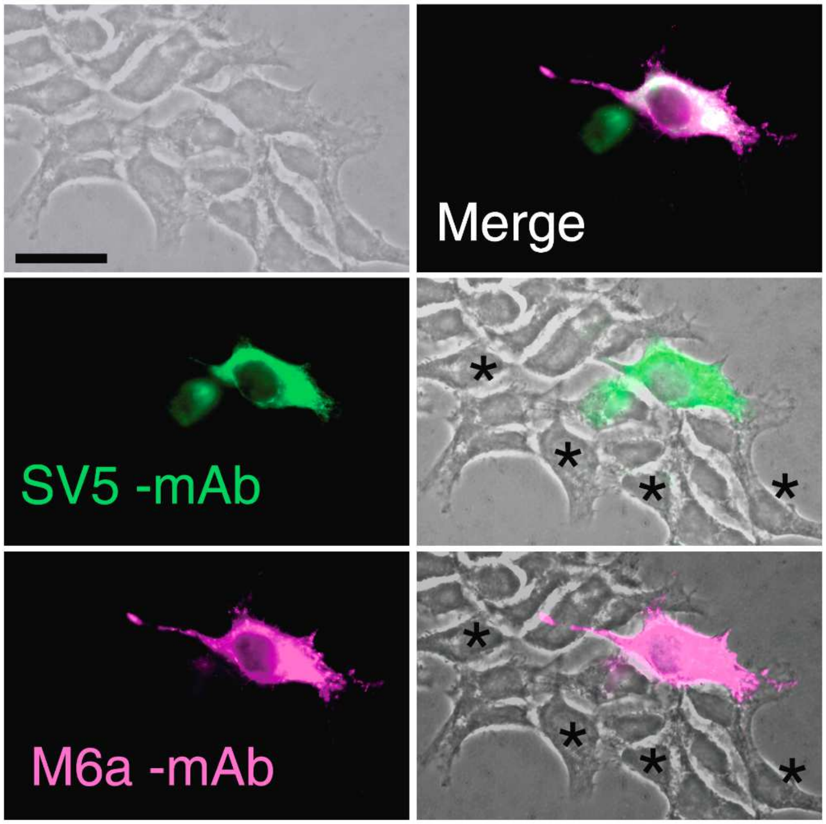

Supplementary Figure S1: HEK293 cells transtiently transfected with pBig plasmid containing M6a-loops. Cells were transiently transfected and 24 hours later they were fixed and labeled under a non-permeabilized condition with M6a-mAb (in magenta). Then cells were washed, permeabilized with Triton-X100, and labeled with Sv5-mAb (in green). Images were taken using a Nikon Eclipse TE2000-U inverted microscope (60X). Black asterisks represent non-labeled intact HEK293 cells (phase contrast). Scale Bar: 30 µm.

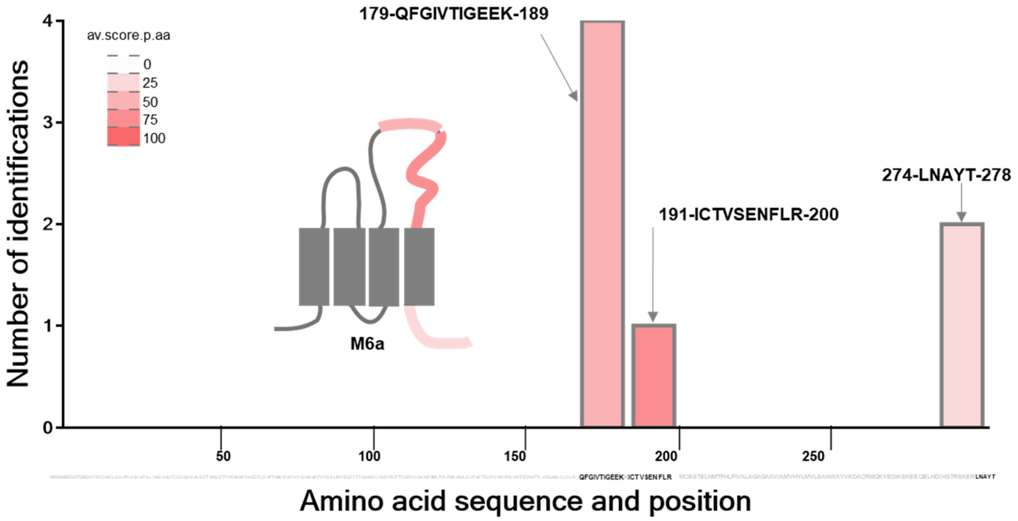

Supplementary Figure S2. M6a sequence coverage by TMT/MS. The graph shows the total number of identified peptides versus the amino acid sequence of M6a and its position. Highlighted is the amino acid sequence of the identified peptides with their corresponding position in M6a protein. Two peptides correspond to the large extracellular loop and one peptide corresponds to the C-tail of M6a. The *av.score.p.aa* represents the intensity of each peptide identified in the TMT/MS.

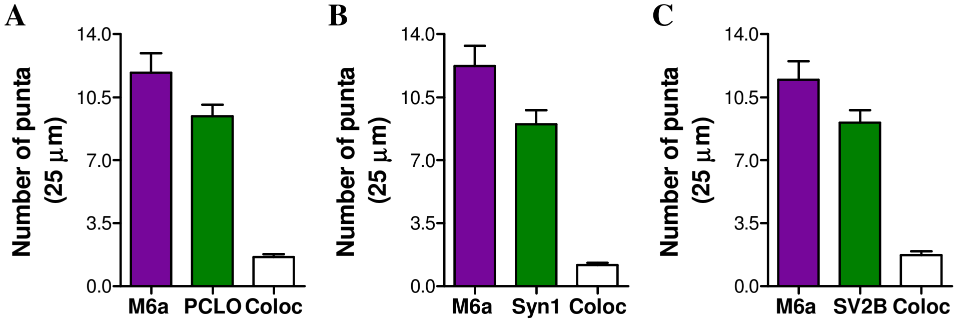

Synaptic Puncta (25 µm dendrite)

	Magenta	Green	Colocalized	Cells (n)
**M6a/Pclo**	11,87 ± 1,069	9,451 ± 0,638	1,632 ± 0,161	34
**M6a/Syn1**	12,24 ± 1,124	9,000 ± 0,799	1,182 ± 0,135	33
**M6a/Sv2B**	11,48 ± 1,053	9,097 ± 0,694	1,736 ± 0,218	24

Supplementary Figure S3: Quantification of colocalization between M6a puncta and PCLO puncta or Syn1 puncta or SV2B puncta by Puncta Analyzer (ImageJ). Hippocampal neurons were seeded at low density (7000 cells/well) and cultured for 14 days. Neurons were labeled for endogenous M6a (magenta) and endogenous piccolo (A), synapsin 1 (B), SV2B (C) (green). The representative image for each condition is shown in Figure 5A-C. The plots correspond to one representative experiment of a total of three experiments. Three segments per neuron were measured and averaged. Each bar in the graph represents the mean per neuron. The table shows the average of synaptic puncta ± SEM and the number of cells quantified for each condition.
